# Recent Advances in Nucleic Acid-Based Electrochemical Sensors for the Detection of Food Allergens

**DOI:** 10.3390/s26010263

**Published:** 2026-01-01

**Authors:** Simone Fortunati, Shaista Nazir, Marco Giannetto

**Affiliations:** 1 Department of Chemistry, Life Sciences and Environmental Sustainability, University of Parma, Parco Area delle Scienze 17/A, 43124 Parma, Italy; shaista.nazir@unipr.it (S.N.); marco.giannetto@unipr.it (M.G.); 2 Biostructures and Biosystems National Institute (I.N.B.B. Consortium), Viale delle Medaglie d’Oro 305, 00136 Roma, Italy

**Keywords:** electrochemical biosensor, food allergen, genosensor, aptamer

## Abstract

Food allergies represent a growing public health concern, requiring analytical methods capable of detecting trace levels of allergenic ingredients in increasingly complex and processed food matrices. In recent years, nucleic acid-based electrochemical sensors have emerged as a powerful alternative to protein-targeting assays, offering improved stability and sequence specificity, as well as compatibility with portable, low-cost sensing platforms. This review provides a comprehensive overview of nucleic acid-based sensing strategies developed for detecting either allergen proteins or nucleic acids related to allergenic species. Particular attention is given to the methodological approaches implemented, which for DNA detection include sandwich-type designs and DNA switches, while for protein detection rely on aptamer-based assays in a labelled or label-free setup. The review also discusses the impact of pre-analytical steps, such as nucleic acid extraction and PCR-based amplification, on assay reproducibility, cost and applicability at the point of need. Although significant improvements in analytical performance have been achieved, challenges remain in terms of simplifying workflows, standardizing methods, validating them on a large scale, and developing continuous monitoring schemes for timely intervention. The review highlights emerging opportunities, including multiplexed detection platforms, robust extraction protocols, and the harmonization of allergen thresholds, which are key to supporting the practical implementation of nucleic acid-based sensors.

## 1. Introduction

Food allergies represent a significant public health concern, with increasing interest fueled by their rising incidence year after year. In the US, it has been reported that approximately 1 in 13 children display symptoms of a food allergy, while the figure for the adult population stands at 10.8%. Data based on clinical history or positive food challenges suggest that the prevalence of food allergies in Europe increased from 2.6% between 2000 and 2012 to 3.5% between 2012 and 2021 [1]. Sensitized individuals suffer severe and, in some cases, life-threatening reactions as a result of ingesting allergens associated with food consumption or protein inhalation. Such reactions stem from type I hypersensitivities, which trigger the cross-linking of the immunoglobulin isotype E (IgE) on the mast and/or basophil cell surfaces. Additionally, reactions can occur via non-IgE-mediated mechanisms, as is the case with gluten allergy in celiac disease [2]. The clinical manifestations resulting from these reactions are often unpredictable and can range from mild to severe, with potentially fatal outcomes. Due to the lack of comprehensive knowledge of the mechanisms that ultimately lead to allergic events, it is difficult to predict how severe a reaction to a food allergen will be [3]. Nevertheless, certain factors are known to play a significant role, including sensitivity to the allergen, age group, and country of origin [4]. However, as Monaci and coworkers highlighted in their review, the allergen’s food matrix and processing must also be taken into account [5]. Indeed, both thermal and non-thermal food treatments modify protein structure affecting solubility, thereby altering the immunological properties of allergens [6].

Periodically, new insights into reaction mechanisms emerge, enabling the identification of novel allergenic proteins and their functions. These proteins are listed in the official WHO/IUIS allergen nomenclature database, “allergen.org”, which, as of November 2025, contains more than 1100 allergens. Around 35% of these are listed as food allergens, with the most common 410 allergens found in the eight food families known as the “Big 8”: fish, crustaceans, milk, eggs, peanuts, tree nuts, wheat, and soybeans. Together, these account for approximately 90% of the allergic reactions, making them the most widely studied [7].

The growing efforts of the scientific community to find cures for food allergies have recently led to the FDA approving the first drug for treating peanut allergy. This is likely to pave the way for similar therapeutics to be approved. In addition, desensitization and remission can in some cases be achieved as an effect of immunotherapy [8], although the success rates of these approaches are variable [9,10]. Therefore, food allergy management still primarily relies on adopting allergen-free diets and treating acute symptoms, which has a significant impact on patients’ lives and on the clinical system. Furthermore, adherence to a strict diet by sensitized individuals can be affected by hidden allergens. These undeclared allergens usually result from cross-contamination events that can occur at any stage of the food production process. Examples include shared transportation vehicles, storage space, and equipment in the food industry, as well as in restaurants and homes [11]. Furthermore, other possible sources of food allergen exposition have recently been identified, including emerging allergens, novel foods, and food contact materials [12]. This has prompted the food industry to introduce precautionary allergen labelling (PAL), in the form of statements such as “may contain”, to safeguard consumer wellbeing. However, PALs are adopted on a voluntary basis, resulting in inconsistent wording that can lead to misconceptions and a poor understanding of the potential risks by consumers [13,14].

In this particularly challenging scenario, analytical techniques that enable the detection of allergens in food are crucial for ensuring compliance with labelling requirements and enabling consumers to adhere to a risk-free diet.

## 2. Regulatory Frameworks for Food Labelling

In the absence of effective treatments for food allergy symptoms, strict regulation is essential to ensure the safety of those affected. However, when dealing with food allergens, it must be considered that these hazardous substances are, in fact, desirable nutrients for most consumers. Therefore, an approach aimed at removing the allergens from the food is neither viable nor desirable. In this context, food labelling represents the only method that can be routinely implemented to enable the sensitive population to adopt an allergen-free diet.

Despite the global scale of the issue, different countries have introduced significantly different regulations [15]. This lack of harmonization even affects major aspects, such as which species are classified as allergens.

In 1963, the Food and Agriculture Organization and the World Health Organization jointly established the commission responsible for the Codex Alimentarius, a collection of internationally recognized standards, guidelines, and codes of practice relating to food safety and quality [16]. The Codex features the “General Standard for the Labelling of Pre-packaged Foods”, which provides a list of the eight major food allergens that require labelling. Additionally, it includes a list of seven ingredients that may be considered for mandatory labelling. The purpose of this guideline is to provide an international standard on which countries can base their regulatory frameworks for food labelling requirements.

The European Union committee began the process of harmonizing food labelling through an administrative act dating back to 1979, and in 2003 it passed the first directive concerning foodstuffs [17]. In 2011, the Commission passed the Regulation No. 1169/2011 on the provision of food information to consumers. Annex II of this regulation contains a list of the 14 allergens currently considered for labelling purposes. These are: cereals containing gluten; crustaceans; eggs; fish; peanuts; soybeans; milk; nuts, including hazelnuts, almonds, walnuts, cashews and pecan nuts; celery; mustard; sesame seeds; sulphur dioxide and sulphites; lupin; molluscs. Mandatory labelling is required for all foodstuffs containing one of the allergens listed, which must be in an emphasized typeset. However, no concentration limits are specified, except for sulphur dioxide and sulphites, which require labelling if they exceed a concentration of 10 mg/kg or 10 mg/L. Specific exemptions for many allergens are listed in Annex II, primarily for highly refined products in which allergenic proteins are no longer present. It is worth mentioning that the Regulation specifically states that the list contained in Annex II is subject to re-examination and updating.

In the United States, the Food and Drug Administration (FDA) is responsible for overseeing food safety. The regulatory framework comprises the Federal Food, Drug, and Cosmetic Act (FD&C Act), the Food Allergen Labeling and Consumer Protection Act (FALCPA), and the Food Safety Modernization Act (FSMA), all of which protect consumers against allergens in food. Together, these define the requirements for allergen labelling on food products [18]. In the US, mandatory labelling is only required for the nine major food allergens: eggs, fish, milk, crustacean shellfish, peanuts, tree nuts, wheat, soybeans, and sesame. Also in this case, no concentration thresholds are defined, meaning that the presence of an allergen alone is sufficient to require product labelling.

Other countries base their allergen labelling requirements on the Codex General Standard for the Labelling of Pre-packaged Foods. However, the recognized allergens often vary in number and type.

Furthermore, some countries enforce threshold limits for allergen concentrations, below which labelling is not mandatory. For example, Japanese regulations have set this limit at 10 ppm of soluble allergenic protein since 2002 [19]. Switzerland requires PAL in the form of “may contain traces of” or “traces of” for allergen amounts exceeding 1000 ppm [20]. Aside from these two examples, threshold limits are mostly lacking in countries’ regulations, for both mandatory allergen declarations and PAL. In an effort to provide a harmonized concentration limit, the European consortium Global Allergy and Asthma Excellence Network (GA^2^LEN) has proposed adopting a threshold of 0.5 mg of protein per 100 g of processed food for the voluntary declaration of food allergen traces in processed food [21]. This harmonized approach would benefit consumers and suppliers alike, while also providing a much-needed threshold to establish the fitness-for-purpose of analytical techniques.

## 3. Analytical Techniques for Food Allergen Detection

As previously discussed, allergic reactions are triggered by specific proteins found in allergens. The most straightforward analytical approach is therefore to detect these proteins in food matrices. In this context, the most popular approach consists of adopting enzyme-linked immunosorbent assays (ELISA), which rely on antibody receptors to bind specifically to the allergen protein. The popularity of ELISA tests, as demonstrated by their widespread commercialization, can be attributed to their unique advantages, such as speed, high throughput, and the simplicity of the analytical workflow, which does not require highly skilled personnel [22]. ELISA tests have been developed for all the “big 8” allergens, displaying a fit-for-purpose sensitivity [23,24,25,26]. However, a major drawback of these techniques is the potential for cross-reactivity between structurally similar allergens, which can lead to false positive results and make accurate allergen identification challenging. Examples include the cross-reactivity of pecan proteins for walnut allergen detection [27], mollusc allergens and mite allergens [28], and edible insects in novel foods with crustacean allergens [29]. The context of allergen detection also raises issues related to food processing. For example, it has been observed that the ability of ELISA tests to detect the presence of thermally labile compounds is affected by the high-temperature treatment of foodstuffs. This can lead to a dangerous underestimation of the protein content [30,31].

Significant improvements can be achieved using mass spectrometry-based approaches which can detect either the entire allergenic protein, provided its molecular weight falls within the analyser’s limits, or, more commonly, specific peptide fragments obtained by enzymatic digestion [32,33,34]. In this context LC-MS techniques enable highly selective allergen detection at trace levels [35]. In fact, targeting the amino acid sequence avoids interference caused by changes in the protein’s secondary and tertiary structures, which commonly affect ELISA methods. Furthermore, the characterization of the sequence, enabled by the availability of dedicated databases, can determine modifications undergone by allergen proteins caused by either enzyme activities or thermal treatments [36,37]. Adopting enzymatic digestion approaches also reduces the complexity of processed food matrices, although protein extraction remains challenging in terms of reproducibility. LC-MS-based approaches notably offer the possibility of multiplexing, enabling the simultaneous monitoring of different signature peptides relevant to food allergens [38,39]. Despite the strengths of these powerful techniques, some challenges still limit their implementation. Sample pretreatment typically involves several time-consuming steps, and alongside extraction efficiency and digestion yields, these steps may affect reproducibility and recovery rates [40,41]. Additionally, implementing these techniques requires expensive instrumentation, specialized laboratories, and trained personnel, hindering their application in point-of-need contexts.

An alternative strategy for detecting allergen proteins or relevant fragments is provided by genomic approaches, which aim to detect DNA sequences related to the presence of specific allergens in food. Unlike proteins, which are susceptible to food processing and biological variations, DNA exhibits a higher thermal stability and remains unaffected by phenotypic changes [42]. In this context, PCR-based methods represent the gold standard as they enable the amplification and detection of DNA sequences with high selectivity and sensitivity. Technologies such as quantitative PCR (qPCR) [43,44] and droplet digital PCR [45,46] allow for the target DNA to be quantified in a comparatively short analysis time [47]. It is worth noting that, by carefully selecting the sequence targeted by the PCR method, it is also possible to detect an allergen that is common to several species in different food matrices. This was demonstrated by Cau and coworkers with respect to the detection of fish allergens in food [48]. Thanks to the simple analytical workflow and established technologies, several PCR methods have been developed for the detection of allergens in food, including commercial kits [49,50,51]. In addition, multiplexing can be achieved by introducing multiple sets of primers that target specific regions of different allergens or, in some cases, the same allergen. This improves the sensitivity and specificity of the detection [52,53,54,55]. Different regions can be amplified by carefully designing the probe sets, and discrimination can be carried out by exploiting different fluorophore tags for each target or based on the melting temperature of the amplicons generated. However, PCR-based methods rely on expensive, non-field-deployable instrumentation, and trained personnel are required to operate the equipment and carry out the sample treatment steps.

In this complex and challenging scenario, biosensors represent a versatile analytical tool, offering reliable, cost-effective and sensitive platforms [56,57]. These self-contained devices exploit a recognition mechanism based on biological interactions to harness specificity and can rely on different approaches to generate the analytical signal. Biosensors can be categorized according to their transduction mechanism as optical, electrochemical, electrical, piezoelectric, etc. Each category exhibits its own advantages and limitations; however, electrochemical transduction is one of the most widely implemented due to its ability to achieve impressive sensitivity and the possibility of miniaturizing the acquisition devices. This results in inexpensive, field-deployable solutions that enable non-experts to test food products at the point-of-need, thus improving food safety [58]. In the context of food allergen detection, electrochemical biosensors enable the use of biorecognition elements based on antibodies, oligonucleotides, aptamers, and molecularly imprinted polymers, allowing for the detection of proteins as well as oligonucleotide sequences [59,60,61]. Although some degree of sample treatment is usually required, this is less labour-intensive than other techniques such as LC-MS [62,63]. An example showcasing the features offered by electrochemical sensors was reported by Cho and coworkers in 2025. The group developed the “iEAT2” (integrated Exogenous Allergen Test 2) multiplex testing platform, complete with a kit for rapidly and reliably processing of samples. This enables highly sensitive detection of gliadin, Ara h1 and ovalbumin in as little as 15 min. It is worth noting that electrochemical sensors aimed at detecting food allergen proteins can be affected by the same limitations reported for ELISA with regard to thermal treatment performed during food processing.

**Scheme 1:**
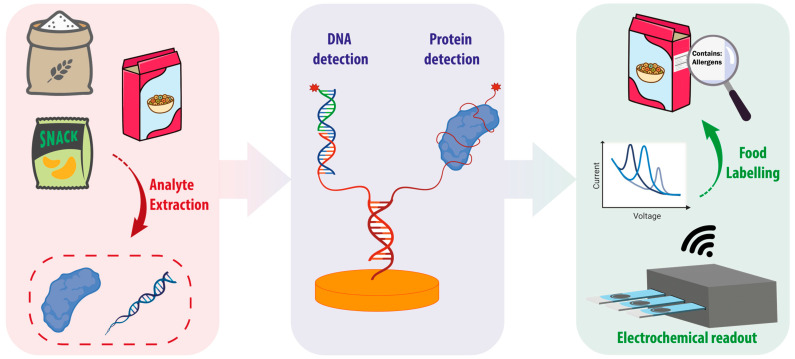
Schematic representation of the analytical workflow aimed at achieving food allergen labelling using nucleic acid-based sensors for the detection of allergen DNA or proteins.

The present review focuses on the implementation of nucleic acid-based bioreceptors to detect allergen-related proteins and DNA in foodstuff (Scheme 1), taking into account studies published in the literature over the last 15 years.

### 3.1. Nucleic Acid-Based Sensors for Allergen DNA Detection

**Figure 1:**
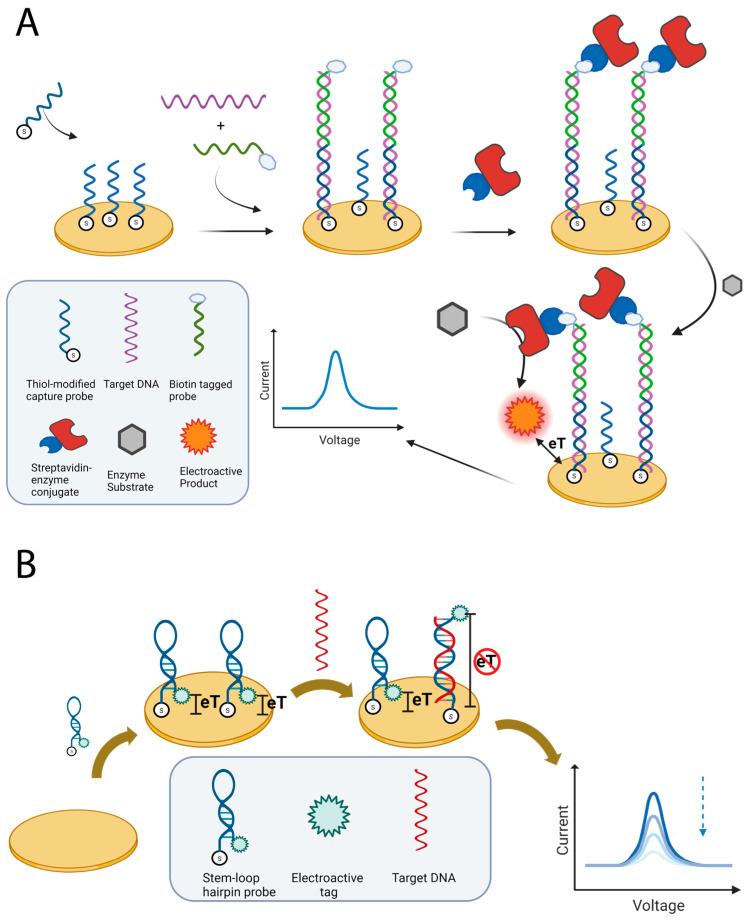
Schematic representations of approaches for detecting allergen DNA. (**A**) The sandwich approach involves immobilizing a capture probe on a gold SPE and using a biotin-labelled signalling probe for detection. The biotin enables enzyme linking, converting a suitable substrate into an electroactive species. (**B**) The DNA switch approach uses a stem-loop probe that is immobilized at one end and carries an electroactive tag at the other end. This positions the tag close to the electrode. When the target DNA binds, the loop opens, increasing the tag–electrode distance and reducing the signal. Figures created in https://BioRender.com, accessed on 19 December 2025.

Electrochemical genosensors have emerged as a powerful analytical tool for determining allergen-specific DNA sequences. These biosensors exploit the complementarity of nucleic acid sequences to assemble specific architectures that allow for target detection. Typically, a single-stranded sequence, commonly referred to as capture probe (CP), hybridizes the target oligonucleotide. The molecular recognition event is then translated into a measurable electrochemical signal using various approaches. An additional single-stranded sequence, called signalling or reporter probe (SP or RP) that is labelled with the desired tag can be used to develop the electrochemical signal [64]. This is achieved by forming a sandwich complex, in which both probes hybridize to separate portions of the target (Figure 1A) [65,66,67]. Another approach involves implementing a molecular switch based on a hairpin probe. In this case, a single-stranded probe, labelled at one end with an electroactive tag, undergoes a conformational rearrangement upon target binding, modulating the signal response by modifying the tag-electrode distance (Figure 1B) [68,69]. Both approaches exploit the predictable base-pairing interactions and structural stability of nucleic acids to provide high sequence selectivity and adaptability to various electrode designs and labelling strategies. This versatility has enabled the development of reliable nucleic acid-based genosensors for allergen detection within complex food matrices (Table 1).

The early implementation of this concept predominantly adopted the sandwich configuration, due to its straightforward design and versatility in terms of labelling. In 2014, this strategy was adopted by López et al., who developed a genosensor for an 86-mer DNA fragment encoding a portion of the peanut allergen Ara h 2 [70]. The sensor exploited a thiolated DNA capture probe immobilized on a gold screen-printed electrode (SPE), to which a biotinylated signalling probe hybridized in an adjacent region of the target, completing the sandwich structure. The resulting complex was labelled with a streptavidin–horseradish peroxidase (HRP) conjugate to enable the enzymatic generation of an electroactive product, thus allowing for the hybridization event to be monitored using differential pulse voltammetry (DPV). The authors focused on forming a mixed self-assembled monolayer (SAM) for the formation of the sensing layer, optimizing the receptor-to-spacer ratio using a design of experiments approach. This highlighted the crucial function of the spacer in preventing steric and electrostatic hindrance between the densely packed DNA strands. Under optimized conditions, the genosensor achieved a limit of detection of approximately 10 pM within a linear range spanning from 0.05 to 50 nM, ensuring high selectivity. Despite highlighting the importance of the nanoscale organization of the probes for analytical performance, the sensor was not tested for its ability to detect allergen DNA in real samples. Also in 2014, Martín-Fernández et al. reported a sandwich assay based on gold SPE, which took a similar approach to targeting a structured 33-mer fragment of gliadin DNA [71]. However, this assay included a PCR pre-amplification step to improve hybridization efficiency by amplifying short targets. The sensor was based on the use of a thiolated capture probe that was immobilized on a gold electrode and hybridized with a FITC-labelled signalling probe. Anti-FITC–HRP conjugates enabled amperometric detection by HRP-catalyzed oxidation of TMB/H_2_O_2_. The assay achieved a limit of detection of 0.3 nM with a linear range of up to 50 nM. The assay showed high sequence specificity with minimal cross-recognition toward related cereal DNA. Although the sensor was tested for the detection of genomic DNA extracted from cereal flour samples, the required PCR pre-amplification step increases assay times and is subject to the aforementioned limitations of this technique. In a subsequent work, the same group used the previously developed assay to extend its applicability to highly processed food matrices, focusing on thermally treated wheat products [72]. The working mechanism was not modified, and the authors reported an LOD of 0.001% for wheat (≈0.9 ppm gluten), comparable to the regulatory labelling threshold. Furthermore, the authors tested the gluten detection method on flour samples, as well as on highly processed foods such as cookies, beer, and snacks. The authors observed a good correlation with the results obtained using ELISA, which was conducted by an independent laboratory. In 2008, Bettazzi et al. developed a disposable electrochemical DNA-array platform for the simultaneous detection of the hazelnut allergens Cor a 1.03 and Cor a 1.04 [73]. The assay was based on a sandwich hybridization scheme in which a thiolated capture probe, which was immobilized on gold SPEs, hybridized with unmodified PCR amplicons and a biotinylated signalling probe. The latter was then labelled with a streptavidin–alkaline phosphatase conjugate, and the resulting α-naphthol product was detected using DPV. The system exhibited detection limits of 0.3 nM for Cor a 1.03 and 0.1 nM for Cor a 1.04, and was successfully applied to detection of hazelnut DNA in extracts from a variety of commercial foodstuffs. The results showed good agreement with those obtained using a commercial ELISA test. Notably, the array format enabled the simultaneous detection of Cor a 1.03 and Cor a 1.04, highlighting discrepancies in the results from some real samples which revealed the presence of only one allergen. This emphasized the need for simultaneous detection. To accelerate the analysis time, in 2017 Montiel et al. coupled Express PCR with magnetic microbeads to detect the hazelnut allergen Cor a 9 [74]. Allergen determination was achieved by assembling a sandwich complex on microbeads and using HRP-based amperometric detection on screen-printed carbon electrodes. The genosensor achieved a limit of detection of 0.72 pM, detecting as little as 20 pg of genomic DNA, within 90 min. Although it still required PCR-based preamplification, this configuration reduced analysis time and improved sensitivity. It is worth highlighting that, although the analysis time was reduced when compared to other techniques requiring more than two hours [70,71,72], 90 min does not meet the demand for rapidity when applied to the continuous monitoring of food allergens. In this context, Lab-on-a-Chip solutions integrating isothermal amplification techniques have been proposed to enable rapid, cost-effective, highly sensitive testing, and reduce amplification complexity [75]. While microfluidic devices based on LAMP or PCR amplification integrated with optical transduction for the detection of allergens have been reported [76,77], to the best of our knowledge electrochemical transduction has not yet been integrated with these technologies for allergen testing.

In an effort to overcome the limitations associated with the inclusion of a PCR-based preamplification step, several approaches have been adopted to achieve amplification-free detection. In this context, Pereira-Barros and coworkers devised in 2019 a sandwich electrochemical genosensor capable of detecting Sola l 7 DNA sequence in genomic extracts without amplification [78]. The assay used RNA-based capture and reporter probes, with the DNA/RNA heteroduplex sandwich assembled on magnetic microbeads. The labelling strategy relies specifically on the use of antibodies (AbRNA/DNA) that recognize DNA/RNA heteroduplexes as well as secondary antibodies labelled with HRP. The enzymatically generated electroactive compound was detected using amperometric measurement after the microbeads were confined magnetically on a SPE. This approach enabled the detection of the tomato allergen sequence with a limit of detection of 0.2 pM and a linear range spanning from 0.8 to 50 pM. The genosensor enabled the direct analysis of non-fragmented genomic DNA extracted from tomatoes (seeds and peel), proving selectivity against corn samples extracts. Furthermore, the amplification-free strategy allowed for the hybridization of the genomic extracts to be performed directly in just 30 min. However, AbRNA/DNA-HRP labelling required an additional 60 min. Building on this, in 2023, the same group developed a PCR-free genosensor for the Sin a 1 mustard allergen using an analogous DNA/RNA heteroduplex sandwich approach [79]. This biosensor achieved a higher limit of detection of 3 pM for the synthetic Sin a 1 target, compared to the genosensor previously developed, with a corresponding linear range from 0.01 to 2 nM. However, the genosensor reduced the total assay time to 75 min as well as the necessary amount of genomic DNA to 50 ng. Real sample analyses were performed by extracting the genomic DNA from yellow radish, hazelnut, and yellow mustard seeds. Good sensitivity and selectivity for Sin a 1 were obtained, confirming the suitability of RNA-mediated, amplification-free genosensors for food analysis.

Although sandwich approaches are predominantly used to develop electrochemical genosensors for detecting allergen DNA, many efforts have focused on implementing genosensors that exploit the conformational switching of a hairpin receptor. The latter consists of a single self-complementary stem–loop probe that adopts a closed conformation in the absence of the target and rapidly switches to an open conformation upon hybridization with target DNA. By functionalizing the hairpin with an electroactive tag, the conformation switch modifies the distance between the tag and the electrode surface, thereby modulating the output signal.

In 2012, Sun et al. described using this approach to design a stem–loop DNA biosensor for detecting of peanut allergen Ara h 1 [80]. The probe was modified with a thiol at one end for immobilization on gold SPEs and with a biotin tag at the other end to hinder the electron transfer. Electron-transfer resistance between a redox species in solution ([Fe(CN)_6_]^3−^/[Fe(CN)_6_]^4−^) and the electrode surface was impeded by the biotin tag, and this effect was monitored using electrochemical impedance spectroscopy (EIS). The sensor demonstrated excellent sensitivity, achieving a LOD of 0.35 fM, and a wide linear range of 1 fM to 0.1 μM. Furthermore, this method was successfully used to quantify Ara h 1 in peanut-milk beverage extracts in only 30 min, without the need for pre-amplification steps. In a subsequent work, Sun and coworkers implemented the stem–loop receptor for Ara h 1 onto an SPE modified with a multilayer graphene–gold nanocomposite [81].The latter possesses superior electron-transfer efficiency, enabling amplification of the output signal and reducing the LOD to 0.041 fM with a linear range from 0.1 fM to 0.1 pM. Once again, the sensor proved capable of detecting peanut genomic DNA extracted from processed peanut milk beverages, with recovery rates ranging from 86.8% to 110.4% in spiked samples. Finally, in 2015 the same group modified glassy carbon electrodes with a chitosan-multiwalled carbon nanotube nanocomposite, onto which spongy gold films were electrodeposited. In this case, the Ara h 1 stem–loop receptor tagged with biotin interacted with a streptavidin–HRP. Upon switching to an open conformation, the enzyme was released from the electrode surface, allowing it to catalyze hydroquinone oxidation [82]. Including an enzymatic signal amplification step enabled the LOD to be reduced further to 0.013 fM, with a linear range of 0.0391–1.25 fM. The Ara h 1 allergen protein was quantified in genomic DNA extracts from peanuts obtaining good agreement with the real-time quantitative PCR analyses.

A mixed approach leveraging a hairpin switch mechanism and a sandwich complex formation was reported by Martín-Fernández et al. in 2015 to determine gliadin [83]. The authors used a receptor with a stem-loop conformation that was not modified with a label, and a signalling probe tagged with biotin for subsequent enzyme labelling. This approach builds on a previous study by the same group, whose mechanism involved only the formation of the sandwich complex [71]. The genosensor displayed similar performance to that previously obtained, with a linear range of 5 to 50 nM and an LOD of 1 nM. However, using a stem-loop configuration for the receptor strand increased selectivity enabling discrimination between closely related sequences. To demonstrate this, the authors successfully discriminated the DNA sequence encoding wheat gliadin from those encoding barley and rye prolamins. The authors argue that the modest reduction in sensitivity obtained by using a stem-loop receptor is outweighed by the significant increase in selectivity.

Interestingly, to the best of our knowledge, artificial DNA mimics have rarely been used to develop electrochemical detection of allergen DNA. In the field of food authenticity, our group has developed electrochemical genosensors that utilize probes based on peptide nucleic acids (PNA) to detect genetically modified soy. Although these probes are more expensive than analogous DNA sequences, the superior performance of PNA greatly enhances the analytical performance of the oligonucleotide detection. In fact, their implementation as receptors directly bound to single walled carbon nanotubes-modified SPEs enabled to reach a LOD of 64 pM [66]. Furthermore, their implementation on magnetic beads for the development of the electrochemical assay enabled to further reduce the LOD to 415 fM [84]. It should be noted, however, that the enhanced PNA-DNA binding affinity may limit sensor regeneration in the context of continuous monitoring. Nevertheless, these examples of the detection of allergen DNA demonstrate the significant potential of integrating artificial DNA mimics as bioreceptors, a field that remains largely unexplored to this day.

**Table 1:** Performance comparison of nucleic acid-based sensors for allergen DNA detection.

Sensing Strategy	Target Allergen	Preamplification	LOD	Linear Response Range	Validation	Reference
Sandwich assembled on gold SPE enzymatically tagged with HRP	Peanut Ara h 2	n.a.	10 pM	0.05–50 nM	Not performed	[70]
Sandwich assembled on gold SPE enzymatically tagged with Anti-FITC-HRP	Gliadin (Wheat)	Yes (PCR)	0.3 nM	1–50 nM	Genomic DNA from cereal flour	[71]
Sandwich assembled on a gold SPE enzymatically tagged with Anti-FITC-HRP	Gliadin (Wheat)	Yes (PCR)	0.001% wheat (≈0.9 ppm gluten)	Not reported	Genomic DNA from processed foods (cookies, beer, snacks and bread)	[72]
DNA-array using a sandwich configuration on gold SPE and ALP enzyme tag	Cor a 1.03/Cor a 1.04 (Hazelnut)	Yes (PCR)	0.3 nM (Cor a 1.03)/0.1 nM (Cor a 1.04)	Up to 20 nM	Genomic DNA from chocolate, soy milk, biscuits, cereals, ketchup, peanut butter, and snacks	[73]
Sandwich assembled on magnetic microbeads with HRP enzyme tag	Cor a 9 (Hazelnut)	Yes (Express PCR)	0.72 pM	2.4–750 pM	Genomic DNA from hazelnut, nut, cashew, pistachio and tangerine	[74]
DNA/RNA heteroduplex sandwich assembled on magnetic microbeads with AbRNA/DNA-HRP labelling	Sola l 7 (Tomato)	No	0.2 pM	0.8–50 pM	Genomic DNA from tomato seeds/peel and corn	[78]
DNA/RNA heteroduplex sandwich approach assembled on magnetic microbeads with AbRNA/DNA-HRP labelling	Sin a 1 (Mustard)	No	3 pM	0.01–2 nM	Genomic DNA from yellow radish, hazelnut, yellow mustard seeds	[79]
Label-free setup based on a stem-loop probe immobilized on a gold SPE	Ara h 1 (Peanut)	No	0.35 fM	1 fM–0.1 μM	Genomic DNA from Peanut-milk beverage extracts	[80]
Label-free setup based on a stem-loop probe immobilized on multilayer graphene-gold nanocomposite	Ara h 1 (Peanut)	No	0.041 fM	0.1 fM–0.1 pM	Genomic DNA from processed peanut milk beverages	[81]
Stem-loop probe labelled with HRP immobilized on spongy gold film electrodeposited on modified glassy carbon electrodes	Ara h 1 (Peanut)	No	0.013 fM	0.0391–1.25 fM	Genomic DNA from peanut	[82]
Hybrid setup using a stem-loop receptor and the formation of a sandwich for HRP enzyme tag	Gliadin (Wheat)	n.a.	1 nM	5–50 nM	Not performed	[83]

n.a. = not applicable.

### 3.2. Nucleic Acid-Based Sensors for Allergen Protein Detection

In the field of allergen detection, electrochemical genosensors are primarily used to identify the DNA of allergen species. However, nucleic acid-based bioreceptors have also emerged as a useful tool for detecting proteins. This process is enabled by specific short-stranded DNA or RNA sequences called aptamers. These are capable of forming high-affinity interactions with small molecules, ions, and proteins [85]. In particular, the interaction with specific protein sites is enabled by the adoption of a specific folding of the single-stranded oligonucleotide. Aptamers are most frequently implemented by covalently binding the single-strand sequence to the sensor’s surface and the binding event with the target protein can then be determined by monitoring various properties of the sensing system using either a labelled or label-free approach. It is worth noting that the aptamer retains its inherent ability to bind a complementary oligonucleotide sequence, thus allowing for remarkable versatility in developing the recognition mechanism. This has paved the way for the implementation of aptamers in electrochemical genosensors in many different fields, including healthcare and food safety [86].

**Figure 2:**
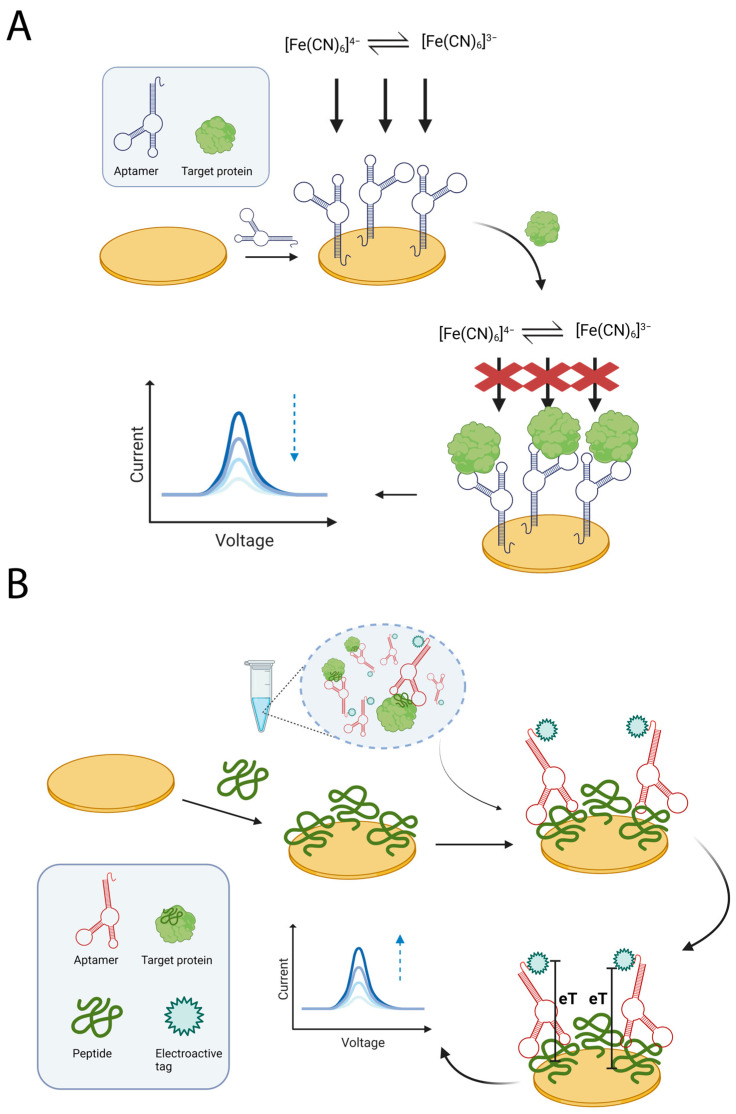
Schematic representations of generic approaches for the detection of allergen proteins. (**A**) A label-free approach based on exploiting an aptamer immobilized on a gold SPE. The binding of the allergen protein to the aptamer hinders the accessibility to the electrode surface by redox species in solution, resulting in a reduced output signal. (**B**) An approach based on competition between the target allergen protein in solution and an immobilized peptide for a labelled aptamer. Binding of the aptamer to immobilized peptide generates a signal that is inversely proportional to the amount of target protein in solution. Figures created in https://BioRender.com, accessed on 19 December 2025.

Unlabelled aptamers have been used in a direct binding configuration on the sensing surface to monitor the changes in sensor surface accessibility due to protein binding (Figure 2A). In 2019, Titoiu et al. adopted this approach to detect lysozyme in wine [87]. Their strategy involved immobilizing a thiolated aptamer on gold screen-printed electrodes, with the [Fe(CN)_6_]^4−/3−^ redox probe signal monitored by cyclic voltammetry. The binding of lysozyme generated steric hindrance, creating a diffusion barrier that decreased the anodic peak associated with the redox probe. The aptasensor proved capable of detecting lysozyme in wine samples at different stages of production, with results consistent with those obtained by HPLC. However, the authors noted that the sensitivity achieved, with an LOD of 0.32 μg/mL, was comparatively low, and that sample preconcentration may be required. This approach may be improved by adopting a more sensitive acquisition technique as reported in 2022 by Svigelj and coworkers [88]. The group developed an aptasensor for gliadin detection using electrochemical impedance spectroscopy to monitor protein binding. Adopting EIS alongside the implementation of gold nanoparticles on the surface of screen-printed electrodes enabled to achieve a good sensitivity, with a LOD of 0.05 μg/mL for gliadin. Validation of the sensor was performed by testing gluten-free beers and gluten-free soy sauce, before and after spiking with gliadin, achieving recovery values between 93% and 105%. Regarding the integration of aptamer receptors with advanced sensing surfaces, Erdoğan et al. developed an electrochemical sensor in 2023 based on the combination of an aptamer and a molecularly imprinted polymer to maximize interactions with the lysozyme protein [89]. Following the covalent immobilization of the aptamer-lysozyme complex on electrodes modified with graphene oxide and gold nanoparticles, the polymer was electrodeposited and the lysozyme was subsequently released under optimized conditions. This nanohybrid receptor was capable of monitoring lysozyme binding using a label-free approach based on the previously described redox probe. An LOD of 3.67 fM was reported with a linear range spanning from 1 fM to 100 pM. However, validation was limited to assessing the recovery values of the spiked allergen proteins in diluted cherry juice, mixed fruit juice and red wine samples. An interesting approach, reported by Jiang et al. in 2021, took advantage of an origami paper-based sensor to detect the Ara h 1 peanut allergen [90]. The aptamer directed to the allergen protein was immobilized on the paper-based working electrode that was modified by the electrodeposition of black phosphorus nanosheets. Microfluidic channels were used for sample loading and for the subsequent sequential folding of the three-dimensional origami chip. The change in the current obtained by the electrochemical reaction of the [Fe(CN)_6_]^3−/4−^ redox probe was then monitored and correlated with the aptamer bioreceptor binding the protein. The sensor achieved a detection limit of 21.6 ng/mL with a wide linear range of 50–1000 ng/mL and demonstrated good recovery rates in spiked cookie dough sample extracts. Furthermore, the developed solution benefits from rapid analysis times of approximately 20 min, cost-effectiveness of the paper-based materials, and a sensor design featuring two independent working electrodes for multiplexing purposes.

Although label-free approaches benefit from inexpensive and minimally modified oligonucleotide receptors, they often display modest sensitivity due to the limited output observed at low analyte concentrations. In contrast, labelled approaches require the receptor oligonucleotide to undergo additional modification to introduce specific electroactive tags (Figure 2B). However, this often improves analytical performance (Table 2).

In this context, Melinte et al. reported in 2023 the development of an aptasensor for Ara h 1 peanut allergen detection in food [91]. The sensor featured a novel electrode material incorporating a bimetallic nanocomposite on graphene oxide. The Ara h 1-selective aptamer was immobilized on this material by coupling of its amino functionality with the carboxy functionalities present on the graphene oxide. Additionally, the aptamer was modified at the other end with a ferrocene label. In the absence of protein, this label remained close to the electrode surface, enabling electron transfer phenomena. However, upon refolding induced by Ara h 1, the label moved away from the sensing surface. The authors then monitored the signal ‘off’ response generated by peanut allergen protein binding using DPV, achieving a LOD of 1.66 nM with a linear concentration range of 5–150 nM. A similar approach was adopted in 2023 by Tertis and coworkers, who achieved gliadin detection by immobilizing a ferrocene-labelled aptamer on a gold nanoparticle-modified screen-printed carbon electrode [92]. Interestingly, in this case, the aptamer displays the label distant from the surface in its non-binding state, and electron transfer is only allowed after binding-induced folding, resulting in a ‘signal on’ detection. The biosensing platform was reported to detect gliadin down to 3.4 ng/L, with an impressive linear range of 10–50,000 ng/L, and its ability to detect the allergen protein was demonstrated in gluten-free flour and breakfast cereals.

The above solutions involve directly immobilizing the aptamer on the sensing surface, followed by capturing the allergen protein. While this approach is straightforward, it can lead to issues related to aptamer immobilization, which could reduce its ability to adopt the folding required for protein binding. Therefore, indirect approaches can be devised to perform the recognition event in solution. In this context, in 2014 the group of Lobo-Castañón and coworkers reported an aptamer-based competitive assay for the detection of gliadin [93]. In this approach, the aptamer is mixed with a sample containing the gliadin target in solution, along with magnetic beads modified with a 33-mer recombinant peptide that competes with gliadin for the interaction with the aptamer. As the interaction with the gliadin in solution is favoured, the assay exhibits signal-off behaviour, decreasing as the amount of gliadin in the sample increases. Based on careful aptamer selection, the best receptor was produced which displayed no cross-reactivity to proteins from maize, soya, and rice. The authors reported an LOD of 0.5 ppb for gliadin, corresponding to 0.5 ppm for gluten, which outperformed several reference methods. In 2015, the same group expanded the aptamer-based assay by including a second aptamer as an alternative receptor for gliadin [94]. This approach integrated a high-affinity aptamer for use with non-hydrolyzed samples and a second aptamer with lower affinity but capable of detecting the target allergen in hydrolyzed samples. This complementary strategy enabled the gluten content to be quantified in complex flour and beer matrices, producing results consistent with those obtained by three external laboratories. Finally, in 2017, the group adapted the assay so that it could be performed directly on the surface of a screen-printed electrode modified with the 33-mer peptide [95]. The assay maintained its competitive approach for gliadin determination and achieved a limit of detection of 0.113 mg/L.

More advanced detection mechanisms exploit the dual functionality of aptamer, which are able to bind both DNA and proteins. On this topic, Xu et al. reported the development of an electrochemical assay whose working principle was based on the displacement of an aptamer triggered by β-lactoglobulin [96]. This was achieved by functionalising an indium tin oxide electrode with gold nanoparticles, which were then used to immobilize a DNA sequence that was partially complementary to the aptamer sequence. Upon displacement of the aptamer by the allergen protein, the DNA sequence was left free to capture a fully complementary DNA strand that was bound to a BiVO_4_ microsphere. The latter mimics the activity of peroxidase and was used to generate the electrochemical signal acquired using chronoamperometry. The authors reported a detection limit of 7 pg/mL, with a wide linear range spanning from 0.01 to 1000 ng/mL. Notably, the ability of the sensor to detect β-lactoglobulin in infant formula was compared with that of a commercial ELISA, demonstrating a good correlation between the two methods with values close to 100%. In 2021, Qiu et al. also devised a different aptamer-based assay for detecting β-lactoglobulin [97]. In particular, the aptamer adopted a stem-loop conformation which, upon binding to the protein target, exposed a nicking site recognized by a specific enzyme. This enzyme then cleaved and released part of the sequence. This DNA was then free to interact with a methylene blue-containing HCR assembly on the surface of a gold electrode, which reduced the mobility of the assemblies and, consequently, the methylene blue signal. Although this platform is considerably more complex than that reported by Xu et al. [96], it achieved comparable performance, with a detection limit of 5.7 pg/mL and a linear range of 0.01–100 ng/mL. In 2025, Liu et al. integrated an aptamer with a DNAzyme to develop a β-lactoglobulin detection assay [98]. In this instance, the aptamer was initially hybridized with a partially complementary strand, which was released upon interaction of the aptamer with the milk allergen protein. This released strand acted as a DNAzyme and was capable of hybridizing with and cleaving a hairpin, thus releasing a secondary strand that was captured on the surface of a gold electrode by a complementary DNA. Finally, a signalling DNA strand hybridized with the secondary DNA strand to form a sandwich assembly. Thanks to its functionalization with a carboxylated reduced graphene oxide sheet decorated with Au, Pt, and a ruthenium-based redox mediator, an electrochemical output was generated. This approach does not require the use of a nicking enzyme and enables β-lactoglobulin to be detected with performance similar to that previously reported. Bai et al. improved the detection of β-lactoglobulin by exploiting a sandwich-like recognition of the allergen protein by two analogous aptamers [99]. In particular, one aptamer was immobilized on a gold screen-printed electrode while the other was co-deposited with a DNA sequence on the surface of a gold nanoparticle. The latter is complementary to a DNA-silver nanocluster template, and the capture of this template on the electrode is monitored by the oxidation current of silver. This approach yielded excellent performance, achieving a detection limit of 0.87 fg/mL and a linear range of 0.001–1000 pg/mL. Furthermore, β-lactoglobulin was successfully detected in fourteen types of food products, with recovery rates ranging from 87.54% to 113.70% in five spiked samples.

Finally, in 2024, our group developed an electrochemical aptasensor designed to study the interaction between lysozyme protein and different anti-lysozyme aptamers reported in the literature [100]. To achieve this, the aptamers were immobilized onto magnetic beads, with biotinylated lysozyme used as the target protein. Subsequent enzymatic labelling with an alkaline phosphatase-streptavidin conjugate enabled the electrochemical signal to be developed. The electrochemical magnetic aptasensor proved well suited to comparing the performance of different aptamers. Surprisingly, the aptamers showed lower affinities than expected and a strong dependence on the assay buffer, as well as poor sequence specificity of the aptamer-protein interaction. These findings highlight the limitations of aptamers as bioreceptors, as reported in other studies [101,102]. Therefore, prior to aptamer implementation, a thorough characterization of the aptamer-protein interaction is required. For this purpose, multi-technique characterization is desirable, including investigation using electrochemical nucleic acid-based assays.

## 4. Conclusions

Considering that food allergies represent a rising global health threat with potentially life-threatening consequences, management currently relies solely on strict dietary avoidance. Since cross-contamination and hidden allergens pose significant risks in processed foods, accurate analytical detection is crucial for verifying food safety, ensuring regulatory compliance, and protecting sensitized individuals from accidental exposure. In this scenario, recent advancements in nucleic acid-based electrochemical sensors highlight their strong potential as innovative analytical tools for detecting food allergens. Compared to conventional protein-based assays, these platforms benefit from the stability of DNA, which is less susceptible to the effects of food processing and biological variability. The development of sandwich-type architectures and hairpin molecular switches has demonstrated excellent sensitivity and high sequence selectivity, as well as compatibility with miniaturized, low-cost electrodes. This enables analysis in complex food matrices.

However, some limitations still need to be addressed, including the reliance on pre-amplification steps, such as PCR, which increase assay time and require specialized equipment, hindering true point-of-need implementation. Even amplification-free platforms often require optimization of nucleic acid extraction workflows, which represent a bottleneck in terms of reproducibility, labour intensity and the ability to address highly processed food matrices. Furthermore, while excellent analytical performance has been reported, validation on real commercial food products remains limited for several approaches.

Looking ahead, several directions appear particularly promising. Integrating amplification-free detection approaches with minimal sample treatment protocols will play a key role in enabling deployment in decentralized settings. The development of multiplexed sensing platforms capable of detecting multiple allergens could provide a more comprehensive understanding of potential contamination events. In this context, technological advancements have been reported regarding the integration of microfluidic platforms in Lab-on-a-Chip devices. These platforms streamline and improve the analytical workflow by combining automated sample treatment, nucleic acid amplification (e.g., PCR and LAMP) and detection in a single device. Given the excellent results obtained with these platforms in the context of food quality monitoring and allergen detection using optical transduction, their integration with electrochemical techniques offers a promising perspective to achieve rapid on-site detection of allergens.

Notably, electrochemical genosensors have recently proven particularly promising in terms of integrating smart acquisition devices with advanced data processing algorithms, e.g., machine learning. While such approaches have not yet been reported in relation to food allergen detection, their implementation represents a promising outlook to improve classification accuracy for allergen contamination in food products.

Finally, closer alignment between sensor development and evolving regulatory needs, including the definition of harmonized threshold values for allergen traces, will be crucial in translating these technologies into practical solutions that support accurate labelling, consumer protection and improved management of food allergies.

## Figures and Tables

**Table 2:** Performance comparison of nucleic acid-based sensors for allergen protein detection.

Sensing Strategy	Target Allergen	LOD	Linear Response Range	Validation	Reference
Label-free setup using a thiolated aptamer immobilized on gold SPE	Lysozyme	0.32 μg/mL	1–10 μg/mL	Spiked wine samples	[87]
Label-free setup using a thiolated aptamer immobilized on gold nanoparticles-modified SPE	Gliadin (wheat)	0.05 μg/mL	0.1–1 μg/mL	Spiked gluten-free beers and gluten-free soy sauce	[88]
Label-free setup using a nanohybrid receptor combining aptamer and MIP immobilized on SPE modified with gold nanoparticles	Lysozyme	3.67 fM	1 fM–100 pM	Spiked cherry juice, mixed fruit juice and red wine	[89]
Label-free setup using origami paper-based platform modified with aptamer-decorated black phosphorus	Ara h 1 (Peanut)	21.6 ng/mL	50–1000 μg/mL	Spiked cookie dough	[90]
Ferrocene-labelled aptamer immobilized on a graphene oxide SPE modified with a bimetallic nanocomposite	Ara h 1 (Peanut)	1.66 nM	5–150 nM	Spiked chocolate cookies, chocolate bar and a chocolate milk-based beverage	[91]
Ferrocene-labelled aptamer immobilized on gold nanoparticle-modified SPE	Gliadin (wheat)	3.4 ng/L	0.01–50 ng/L	Spiked Wheat flour, gluten-free flour and gluten-free breakfast cereals	[92]
Labelled aptamer competition between free protein and a peptide immobilized on magnetic beads	Gliadin (wheat)	0.5 ppb	Not reported	Infant soya formula, cake	[93]
Labelled aptamer competition between free protein and a peptide immobilized on magnetic beads	Gluten (wheat; intact and hydrolyzed)	4.9 μg/L (Gli1 receptor)/0.5 μg/L (Gli4 receptor)	Not reported	Beers, cakes, cereals, snacks, chocolate, vanilla cream, flours	[94]
Labelled aptamer competition between free protein and peptide immobilized on a SPE	Gliadin (wheat)	0.113 mg/L	Not reported	Thickeners, rolled oats and snacks	[95]
Protein-triggered aptamer displacement resulting in capture of a labelled DNA strand	β-lactoglobulin (Milk)	7 pg/mL	0.01–1000 ng/mL	Spiked infant food formula	[96]
Enzymatic cleave triggered by aptamer protein binding activates methylene blue-containing HCR on a gold SPE	β-lactoglobulin (Milk)	5.7 pg/mL	0.01–100 ng/mL	Spiked hypoallergenic formula	[97]
DNAzyme cleave triggered by aptamer protein binding promotes sandwich formation on a SPE	β-lactoglobulin (Milk)	5.4 pg/mL	0.01–100 ng/mL	Spiked infant rice cereal	[98]
Sandwich-like setup using an aptamer immobilized on a gold SPE and a second aptamer enabling immobilization of a DNA-silver nanocluster template	β-lactoglobulin (Milk)	0.87 fg/mL	0.001–1000 pg/mL	Chocolate, Cookie, milk, infant formula, cheese snacks, yoghurt, fruit juice, candy, green tea	[99]

## Data Availability

The original contributions presented in this study are included in the article. Further inquiries can be directed to the corresponding author.

## Abbreviations

The following abbreviations are used in this manuscript:

CP	Capture Probe
CV	Cyclic Voltammetry
DPV	Differential Pulse Voltammetry
EIS	Electrochemical Impedance Spectroscopy
ELISA	Enzyme-Linked Immunosorbent Assay
FITC	Fluorescein Isothiocyanate
HCR	Hybridization Chain Reaction
HRP	Horseradish Peroxidase
LC-MS	Liquid Chromatography–Mass Spectrometry
LOD	Limit of Detection
LOQ	Limit of Quantification
MIP	Molecularly imprinted polymer
PAL	Precautionary Allergen Labelling
PCR	Polymerase Chain Reaction
PNA	Peptide nucleic acid
qPCR	Quantitative Polymerase Chain Reaction
SAM	Self-Assembled Monolayer
SP/RP	Signalling Probe/Reporter Probe
SPE	Screen-Printed Electrode
TMB	Tetramethylbenzidine
